# Recent Advances in Image-Guided Radiotherapy for Head and Neck Carcinoma

**DOI:** 10.1155/2009/752135

**Published:** 2009-07-29

**Authors:** Sameer K. Nath, Daniel R. Simpson, Brent S. Rose, Ajay P. Sandhu

**Affiliations:** Department of Radiation Oncology, Rebecca and John Moores Comprehensive Cancer Center, University of California at San Diego, La Jolla, CA 92093-0843, USA

## Abstract

Radiotherapy has a well-established role in the management of head and neck cancers. Over the past decade, a variety of new imaging modalities have been incorporated into the radiotherapy planning and delivery process. These technologies are collectively referred to as image-guided radiotherapy and may lead to significant gains in tumor control and radiation side effect profiles. In the following review, these techniques as they are applied to head and neck cancer patients are described, and clinical studies analyzing their use in target delineation, patient positioning, and adaptive radiotherapy are highlighted. Finally, we conclude with a brief discussion of potential areas of further radiotherapy advancement.

## 1. Introduction

Recent technological advances in the field of radiation oncology are revolutionizing the management of cancer with ionizing radiation. Through the use of highly conformal techniques, the ability to deliver curative doses to sub-millimeter accuracy is unprecedented to now. In particular, intensity-modulated radiotherapy (IMRT) has had a substantial impact on the management of head and neck carcinoma (HNC), and its use is highly prevalent among radiation oncologists [1]. IMRT allows for the delivery of high doses to target volumes while simultaneously limiting the dose to organs at risk, so that once common toxicities, such as xerostomia, can be limited. However, for this to be achieved, sharp gradients in dose are produced, and therefore small changes in patient or tumor position may have large dosimetric implications. In particular, several studies have demonstrated that patient/tumor motion during IMRT specifically for HNC is clinically significant [2–4]. 

 Image-guided radiotherapy (IGRT) is a novel array of techniques to help minimize the discrepancies due to variations in patient/tumor position. A strict definition of IGRT is the use of images to monitor or modify treatment delivery. However, IGRT can also be divided into three broad categories of image-based innovations: (1) the integration of functional and biological imaging into the treatment planning process to improve tumor contouring (or *target delineation*), (2) the use of various imaging modalities to adjust for tumor motion and positional uncertainty, and finally (3) the adaptation of treatment planning based on tumor response and changes in normal tissue anatomy [5]. The latter form of IGRT, known as *adaptive radiotherapy*, has the potential benefit of avoiding unintended normal tissue toxicity by altering the original treatment plan according to changes that may have occurred during the course of radiotherapy. 

 Treating HNC is often complex, owing to the importance of preserving critical organ functions, such as salivation, speech, and swallowing, that are key factors in determining quality of life after treatment. Since radiotherapy continues to play a central role in the definitive [6–10], adjuvant [11, 12], and recurrent disease [13] settings of HNC, it is likely that these innovations will continue to improve outcomes by minimizing toxicity and maximizing organ preservation. In addition, dose escalation with IMRT may lead to improved local control, which may ultimately extend survival if augmented by improvements in systemic therapies for metastatic disease. Although many of these sophisticated imaging and treatment modalities that employ IGRT are still yet to be proven beneficial in randomized controlled trials, the theoretical benefits of improved disease-control and normal tissue sparing are currently being demonstrated in a variety of peer-reviewed publications, which is the focus of the following review.

## 2. Improved Target Delineation

The first type of IGRT involves the incorporation of new diagnostic imaging modalities into the initial tumor contouring stage of radiotherapy planning in order to more precisely identify areas that should be treated with radiation. Currently, most centers employ CT-based planning, where the patient is simulated in the treatment position and then the targeting of macroscopic and microscopic disease sites is performed on CT-acquired images alone. Although CT-based planning is common for HNC, recent studies have suggested that a large degree of interobserver variability exists in the contouring of the gross-target volume (GTV). Cooper et al. asked eight “expert” physicians to contour the same GTV in 20 patients with supraglottic carcinomas and found that the overlap in contoured volumes was only 53% with CT-alone [14]. As precise tumor localization is of growing importance with increasingly conformal radiotherapies, attention has now shifted to novel forms of imaging that provide additional biological and tumor information that can be included in the planning process in order to clarify areas of tumor burden. 

 A key innovation in this form of IGRT is the use of 18-F-Flurodeoxyglucose (FDG)-PET. FDG is a radiolabeled analog of glucose that is selectively absorbed in tumor cells more than normal tissues, and thus it is useful in distinguishing neoplastic growth in tissues that otherwise appear radiographically normal. As such, FDG-PET has a well-established role in oncology and is commonly used in tumor staging for several cancers, including HNC [15–17]. However, increased interest has now focused on the use of FDG-PET in target delineation for radiation therapy in order to guide the contouring of tumor margins and extended fields. Since most tumor contouring is performed on CT-based images, this is accomplished by using sophisticated software to perform an accurate overlay (or *registration*) of PET and CT images. In this fashion, target delineation can be performed on the fused PET-CT image. Alternatively, some centers are now equipped with hybrid PET-CT scanners that are capable of acquiring both PET and CT scans during a single session [18]. This has the added benefit of imaging the patient while in the treatment position. 

 Research on FDG-PET in HNC has shown that PET-based planning can significantly influence the size of the gross-tumor volume (GTV) that is outlined [19–24], the size of the nodal volume [23, 24], and assist in the detection of nodal metastases not visualized or enlarged by CT criteria [23, 24]. Most studies have found that PET-based planning tends to reduce the GTV, however some studies have shown that PET-based planning can also increase the size of volumes contoured [5, 19]. Furthermore, new clinical evidence from patients treated with PET-CT planning is appearing in literature. Research has shown that PET-CT based planning can lead to excellent local control [18, 25], significant alterations in staging [22], and decreased normal tissue toxicity [18]. In particular, Vernon et al. reported on 42 patients with HNC who underwent PET-CT during planning and were followed for a median of 32 months [18]. A high level of disease control was obtained, and acute toxicities were relatively mild and improved with time. 

 Although the initial results of improved tumor localization through PET-CT planning are optimistic, several areas of concern exist. Guido et al. raise an issue regarding PET-CT planning in a recent study of 38 patients who were planned using PET-CT and CT-alone [26]. These researchers found that although the GTV was reduced in 92% of patients with the addition PET-CT from CT-only-based plans, the planning target volume (which includes areas of microscopic disease and additional margins for error) was not significantly different between the two planning modalities. As such, no clinical advantage would be expected from the combined PET-CT planning. Further research on technical issues such as this will have to be carefully addressed in the future before widespread implementation of these technologies. As of now, FDG-PET has a well-established role for tumor staging, monitoring tumor response, and follow-up of HNC patients. However, the routine use of PET-CT for planning is not yet recommended. 

 FDG-PET is a commonly used radioactive tracer; however several novel tracers are being employed in HNC imaging. Tumor hypoxia is a common occurrence in the tumor microenvironment and has a well-known role in the resistance of tumors to radiotherapy. Furthermore, it is thought that many hypoxia-induced treatment failures can be prevented in part by escalating the dose to hypoxic subvolumes of the GTV. However, this process depends on our ability to accurately identify hypoxic areas and deliver a targeted radiation boost to those localities. Recent advances in PET-based imaging combined with IGRT are now making “hypoxia-directed radiotherapy” possible [27]. [^18^F]-misonidazole (FMISO) is a novel tracer that has been shown to accurately identify hypoxic areas in head and neck tumors [28–30]. In particular, Lee et al. have used FMISO-PET to identify hypoxic subvolumes in 10 HNC patients and subsequently escalated the dose to those areas with a local boost [31]. No outcomes were reported, but the feasibility of the technique has been established. 

A recent study describes the treatment of 20 HNC patients who received routine pre- and mid-treatment FMISO scans in order to determine the effect of tumor hypoxia on patient prognosis [32]. Surprisingly, these results showed that neither the presence nor absence of tumor hypoxia as defined by FMISO was correlated with patient outcome. Although this may suggest that tumor hypoxia is not correlated with patient outcomes, the authors suggest several alternative explanations to this idea, including the notion of tumor reoxygenation during fractionated radiotherapy. Furthermore, a wealth of preclinical and clinical data support the worsening prognosis associated with hypoxia in HNC [27, 33–36]. In any case, further investigation is necessary to ascertain whether the outcomes of HNC can be improved by specifically targeting hypoxic zones. 

 Other non-FDG tracers have also been investigated for their role in HNC patients. In particular, 1-(^11^C)-acetate PET (ACE-PET) has been shown to be a promising tracer for HNC staging and target delineation and may be used to complement FDG-PET [37]. The molecule, 3′-deoxy-3′-^18^F-fluorothymidine (^18^F-FLT), is also of growing interest to HNC management. FLT is phosphorylated by the cytosolic enzyme thymidine kinase-1 (TK1) and is subsequently trapped intracellularly [38]. TK1 activity is increased during DNA synthesis, and thus ^18^F-FLT trapping is a marker of proliferation. Research specifically in HNC has shown that FLT uptake is correlated with decreased survival [39], has good reproducibility [40] and may potentially be useful in determining tumor response to radiotherapy [41]. Finally, similar to FMISO, Cu(II)-diacetyl-bis(N(4)-methylthiosemicarbazone) (Cu-ATSM) is a marker of hypoxia but through an entirely different mechanism [27]. This tracer has also been evaluated in HNC and was shown to provide another feasible approach for hypoxia-directed radiotherapy [42]. However, further research is necessary before the routine implementation of this or other novel PET tracers into daily clinical use.

## 3. Improved Treatment Delivery

The second type of IGRT involves the use of modern imaging modalities to assist in daily patient positioning. Most radiotherapy protocols involve several weeks of sequential daily treatment, and each day the patient needs to be repositioned into the exact position obtained during the initial planning CT. However, often small positioning errors occur, which introduces the possibility for considerable *interfraction* motion. In addition, if the patient is not properly immobilized during a radiotherapy session, there is also the potential for *intrafraction* motion. As six potential degrees of freedom are prone to changing between and during fractions, accurate positioning is an exceedingly complex challenge. 

 Over the years, several unique methods have been devised to address and minimize interfraction and intrafraction motion. Traditionally in HNC, thermoplastic masks composed of a mesh-like grating are placed over a patient's face and secured to the treatment couch in order to immobilize the patient's head during CT-simulation and treatment. The masks have markings on them that allow the radiation therapists to then re-position the patient prior to each fraction with the aid of optical arrays. Various types of masks [3, 43–47] and bite blocks [48] have been employed for HNC patients. However, due to the flexibility of the head and neck region, these immobilization techniques have a potential for considerable setup variability [45, 49–51]. 

**Figure 1:**
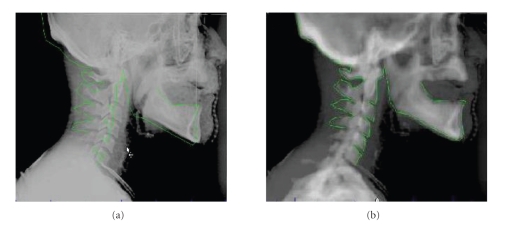
Example of 2D kV image used for verification of patient positioning. A 2D projection was created from the planning CT, and the bony anatomy was contoured (solid line). This image was then overlapped with a kV image taken immediately prior to treatment delivery. The overlay is shown before (a) and after (b) adjustments are made.

 Another common way to verify patient positioning is through the use of two-dimensional (2D) portal film imaging (see Figure 1). This is done using devices attached to the treatment machine that are capable of taking two-dimensional megavoltage (MV) [48, 52, 53] or kilovoltage (kV) [54] radiographs. Typically, this is performed at the beginning of each week of radiotherapy; however newer schemes have been devised for daily kV imaging that are more sensitive to day-to-day interfractional changes [55]. Although these 2D-radiographs are adequate for detecting large positioning errors, they are problematic for a number of reasons. First, they tend to have poor image quality, making it difficult to identify set up inaccuracies [56–58]. Second, they can only visualize bony structures, so changes in soft tissue are not detected using this method. Third, 2D-radiographs are not adequate for detecting rotational movement of the head [49, 59, 60]. 

 As such, recent advances in *three-dimensional* (3D) (or *volumetric*) in-room imaging have offered new solutions to the limitations of conventional patient positioning. One solution that has been proposed involves the use of a conventional CT scanner mounted on a rail system, which is placed in the treatment room and shares a couch with the linear accelerator. This system is capable of taking high-quality, three-dimensional images after patient immobilization in order to verify setup between day-to-day treatments [51, 61, 62]. These images are of higher quality than traditional portal images, and they provide adequate resolution for soft tissue identification. However, the CT-on-rails system does have some distinct disadvantages. First, the addition of a full-size CT gantry into the treatment room can be cumbersome. Second, these systems are incapable of detecting intrafractional motion. Finally, this system introduces the need for movement of the couch between the CT scanner and the linear accelerator, which increases the time of the procedure [63]. 

 Cone-beam CT (CBCT) is another novel form of 3D in-room imaging that can minimize patient positioning inaccuracies. CBCT is a scaled-down version of a CT scanner that is built into the treatment machine. Images taken from a CBCT at the time of treatment can be overlaid on the original planning CT, and specialized software can be used to detect positioning errors with millimeter accuracy [59]. Similar to 2D portal imaging, two types of CBCT exist: MV and kV. CBCT with kV imaging is reported to have better image contrast and smaller signal-to-noise ratios than MV CBCTs [64]. CBCT imaging has been used to correct for interfractional motion in a clinically feasible amount of time [63]. In addition, this technology is being studied for the detection of intrafractional motion, which could potentially be used for improved accuracy as well [65, 66]. Finally, CBCT-based correction has also been used to increase treatment accuracy in the setting of IMRT, thus allowing for larger target doses, while simultaneously sparing healthy tissues [67, 68]. There are concerns; however, about the additional radiation dose delivered with frequent CBCT imaging [69, 70]. In particular, studies have estimated that daily cone-beam CT imaging can lead to an increase of 5.3–6.7 cGy to skin per scan [71] and a total of 300 cGy over an entire treatment course [72]. This may correspond to a 2%–4% increase in secondary malignancies [71]. No long term data on the actual incidence of secondary malignancies is currently available, and continued investigation will have to be performed to address this question. 

 In the past few years, helical tomotheraphy (HT) has become an increasingly popular technique that employs daily volumetric imaging to visualize both patient setup errors and tumor/organ variations [73, 74]. HT combines a 6 MV CT with a therapeutic linear accelerator that is mounted onto a ring gantry. During treatment, the patient is translated through the ring while the gantry continuously rotates, resulting in helical fan beam radiation delivery. The radiation beam is dynamically modifed using a binary multileaf collimator, which allows for IMRT and the creation of highly conformal dose distributions. In addition, using the on-board 6 MV CT scanner, daily image guidance can be performed with the patient in the actual treatment position [75]. Thus, direct target position verification can be achieved prior to radiation delivery [73]. 

 Research on HT in HNC patients has been promising. A prospective evaluation comparing HT to 3D-conformal radiotherapy (3D-CRT) in 60 patients with disease at various anatomic sites found that HT was subjectively equivalent or superior to 3D-CRT in 95% of plans [76]. Furthermore, studies have shown that HT can achieve sharper dose gradients, improve dose homogeneity, and provide better sparing of the parotids than conventional IMRT [77–79] or stereotactic radiosurgery [80]. HT with daily position corrections using MV CT is also safe and easy to implement into a daily clinical routine [74]. Clinical outcomes using HT in HNC patients have also been encouraging and have shown decreased dose, as well as toxicity, to the parotids without compromising survival, locoregional control, and disease-free survival in comparison to conventional and non-HT IMRT approaches [81, 82]. 

 Digital tomosynthesis (DTS) offers another method of 3D in-room imaging for patient setup verification. Similar to CBCT and HT, DTS provides volumetric tomographic imaging; however it works by reconstructing 3D slices from a limited number of 2D cone-beam projections. These images may be of a lower resolution; however advocates of this technology argue that it is comparable to CBCT in terms of imaging quality. Furthermore, since DTS constructs images from a limited number of arcs, it may result in lower cumulative doses, as well as reduced treatment times in comparison to other modalities [83, 84]. These advantages may be of added benefit to pediatric patients, where reduced dose and decreased treatment times are a high priority. 

 Optical methods have also been studied for daily image-guidance [85]. Several groups have reported on systems utilizing in-room cameras for imaging 3D surface reconstructions in real time [86–88]. Others have used specialized cameras with infrared markers for determining target position [3, 89–93]. These systems are reported to detect setup errors with high precision, as well as little setup time. This technology has also been used in combination with in-room radiographic imaging with promising results [44, 94–96]. Unlike other radiographic modalities, optical modalities are noninvasive and do not expose the patient to added radiation dose. In addition, these techniques account for intrafraction motion and can be done in a relatively short amount of time [85]. 

 In conclusion, volumetric (3D) imaging in the HNC setting is superior to conventional 2D portal imaging in many ways. However, the extent to which this technology should be applied is unclear. In particular, the frequency with which 3D imaging for setup verification should be performed is unknown and is the subject of current debate. Some have argued that weekly or biweekly scans are adequate [59], while others have suggested that daily scanning is necessary [97]. Additional investigation will be necessary to clarify these questions.

## 4. Adaptive IGRT

The third broad category of IGRT is called adaptive IGRT (ART). ART is a new, and still evolving, concept with the potential to greatly improve the delivery of radiotherapy. The current standard of treatment planning in radiotherapy involves obtaining an image at the start of treatment. The plan is then generated on that image and delivered to the patient over the course of his/her therapy. We know in head and neck cancer; however, that over the course of the 6–7 weeks of radiotherapy, there can be significant changes in the patient's anatomy based on shrinkage of the primary tumor or involved lymph nodes and loss of overall body weight [98–100]. Applying the original plan to the now altered patient anatomy can lead to increasing the dose delivered to the surrounding normal tissues, including the parotid glands and spinal cord [101–104]. Sparing these normal tissues is an important consideration, because post-radiation xerostomia has a significant impact on quality of life [105–107] and dose constraints regarding the spinal cord and brain stem are always of concern due to potentially devastating consequences. ART allows us to “adapt” the treatment plan in response to the changes that occur so that we can maximally spare these normal tissues while maintaining complete coverage of the tumor volume. 

 A study by Barker et al. examined the rate of tumor regression and the total overall tumor regression by obtaining CT images during treatment 3 times per week over the course of radiotherapy and quantifying the volumetric and geometric changes that occurred [99]. They estimated that the GTV decreased by a median rate of approximately 1.8% per day. The median total GTV decrease was approximately 70% (range 10%–92%) over the course of treatment, and this shrinkage tended to be asymmetric. The parotid volume also decreased by a median of 28.1% and moved medially with a median translation of 3.1 mm which correlated with patient weight loss. Vakilha et al. demonstrated a median reduction of parotid volume of 49.8% and a median medial translation of the parotids of 8.1 mm over the course of treatment [108]. 

 Medial translation of the parotid glands from tumor regression and patient weight loss tend to bring the parotids into higher dose regions and therefore increase the dose to the parotids [101]. In addition, shrinkage of the parotids can result in a much larger percentage of the parotid receiving high doses than anticipated. O’Daniel et al. estimated that the median dose increase to the ipsilateral parotid was 3 Gy, and 45% of patients experienced increases between 5–7 Gy [103]. Though these doses seem small, the parotid is a very radiosensitive tissue and even small changes in dose can have a large impact. Blanco et al. estimated that salivary function decreased at a rate of 5% per 1 Gy increase in mean dose [107]. They also noted that 70% of patients that received a mean dose of greater than 26 Gy to both parotids experienced grade 4 xerostomia. 

 In order to avoid the unintentional overdosing of the surrounding tissues, some investigators have studied re-planning the radiation treatments in response to changes in patient anatomy. Kuo et al. performed a prospective trial in which 10 patients with enlarged lymph nodes were re-planned after delivery of 45 Gy [101]. Twenty-one Gy was then delivered according to the new plan to complete the radiation treatment. The patients were then analyzed to compare the differences between the dose that was delivered after re-planning to the dose that would have been delivered without re-planning. Their results show that the dose to the parotid glands was reduced by approximately 2–4 Gy by re-planning. 

 Hansen et al. performed a retrospective analysis on patients that were re-planned for weight loss or tumor regression [102]. Comparison of the two plans showed that not re-planning led to decreases in target coverage and increases in dose delivered to the surrounding tissues. They found that the dose to 95% of the planned target volume was reduced in 92% of patients in the old plan compared to the new plan (range, 0.2–7.4 Gy). In addition, the maximum dose to the spinal cord was higher in the original plan compared to the new plan in all patients (range 0.2 to 15.4 Gy). The brainstem maximum dose was also increased in 85% of patients (range 0.6–8.1 Gy). 

 Though research in the field of ART is mostly preliminary, it does show promising evidence of an improvement in the delivery of radiotherapy. Though the theoretical benefits of ART are highly desirable, there are still many barriers to overcome before widespread adoption will be feasible. First, it is unclear when and how often re-planning should be done. Would re-planning once be sufficient or would it need to be done more frequently, such as weekly or even daily? Alternately would it be more appropriate to develop defined thresholds that, if met, would necessitate re-planning? Attempts are underway to identify the optimal re-planning schedule, but for now, this schedule must take into account the technical difficulties and the time required to create a new plan. Currently, occasional re-planning can be done, but frequent re-planning would overwhelm the available resources. New technologies such as deformable image registration and automated target delineation in conjunction with higher computational power will be required before widespread adoption of this new strategy can occur.

## 5. Future Technologies

In the future, IGRT will likely continue to expand by incorporating newer and more sophisticated imaging modalities. In this section, we briefly discuss several cutting-edge technologies that are in the early stages of investigation in HNC, including molecular-based CT, high-resolution ultrasound, magnetic resonance imaging (MRI), and proton therapy. 

 Molecular-based CT imaging is a promising modality that may offer several advantages for tumor delineation. As CT is one of the most commonly employed diagnostic imaging modalities in hospitals today, it has widespread availability and convenience of use. However, CT is generally not thought of as a molecular/cellular imaging modality owing to the lack of targeted contrast agents. A recent report by Popovtzer et al. at the University of Michigan at Ann Arbor has described the use of gold nanoparticles that selectively and sensitively target tumor antigens [109]. Using in vitro models of HNC, these researchers demonstrated that the attenuation coefficient for molecularly targeted cells is over 5 times greater than for normal cells. As such, nanotechnology-based CT may improve target delineation by providing more accurate microtumor identification during planning. Furthermore, since CT is easily accessible to most physicians, this technique could be rapidly introduced if proven to be both feasible and efficacious. 

 Aside from CT and PET, several other imaging modalities have also been investigated for their potential role in radiotherapy planning for HNC. High-resolution ultrasound was studied by Wein et al. who demonstrated a feasible method for incorporating ultrasound-based information of the architecture of cervical lymph nodes into the planning CT for target delineation [110]. MRI has also been examined in HNC. A recent study by Gardner et al. has found that MRI fused to the planning CT can decrease the amount of interobserver variation in critical organ and target volume delineation for patients who have intracranial tumor extension, heavy dental work, or contraindication for contrast-enhanced CT [111]. To these authors knowledge, no clinical data has yet been reported. However, based on these preclinical studies, MRI and high-resolution ultrasound may contribute to improved outcomes in HNC patients. 

 Proton therapy is another appealing form of radiotherapy owing to its superior dose distribution properties, which allow smaller volumes of normal tissue to be irradiated than is possible for any photon beam technique. Accordingly, initial clinical experiences of proton therapy in HNC have been encouraging and have shown reduced normal tissue toxicity in sinonasal, nasopharyngeal, and oropharyngeal malignancies [112]. Although long-term efficacy studies are still immature, the preliminary data is encouraging. Furthermore, recent interest in combining proton therapy with modern improvements in image-guidance and dose-localization has arisen. In particular, just as the intensity of photon beams can be modulated in IMRT, the intensity of proton beams can also be modified to produce intensity-modulated proton therapy (IMPT) [113]. Although a mature technique is still unavailable, an offline study in HNC patients has shown that IMPT has a better ability to spare organs at risk and is associated with a significantly reduced risk of secondary malignancy induction in comparison to IMRT with photon beams [114]. The feasibility of combining proton therapy with various forms of IGRT, such as MRI- and kV-based modalities, has also been demonstrated and may lead to a further reduction in normal tissue toxicity when clinical data becomes available [115, 116]. Based on preliminary reports such as this, future proton-therapy research is eagerly anticipated.

## 6. Conclusion

With the advent of highly precise conformal therapies, such as IMRT, the accurate localization and delivery of radiotherapy will be increasingly important in the decades to come. Recent advances in image-guided radiotherapy provide increased tumor localization by improving the identification of areas of tumor burden, by minimizing the effects of patient setup errors caused by intra-/interfraction motion, and by allowing for adaptive replanning to changes that occur in the tumor or patient during long courses of radiotherapy. In doing so, these changes are leading to improvements in the therapeutic ratio, where doses are increased at diseased-sites and minimized at normal tissues. 

 Although the promise of IGRT is great, it is not without its hurdles. Importantly, there are large financial and educational barriers in the initial setup and implementation of new imaging modalities. Furthermore, there is still no existing level I evidence demonstrating the benefit of IGRT over standard radiotherapeutic modalities. Evidence from existing retrospective and nonrandomized studies; however, strongly supports the beneficial role of IGRT. Further research is currently under way, and the results are expected to continue to validate the use of IGRT in the management of HNC patients.
